# Treatment of diarrhea in young children: results from surveys on the perception and use of oral rehydration solutions, antibiotics, and other therapies in India and Kenya

**DOI:** 10.7189/jogh.03.010403

**Published:** 2013-06

**Authors:** Greg Zwisler, Evan Simpson, Melissa Moodley

**Affiliations:** 1 PATH, Seattle WA, USA; 2 IPSOS Healthcare, London, UK

## Abstract

**Background:**

Diarrheal disease is a leading cause of morbidity and mortality among children under five. Although oral rehydration solution (ORS) has tremendous therapeutic benefits, coverage of and demand for this product have remained low in many developing countries. This study surveyed caregivers and health care providers in India and Kenya to gather information about perceptions and use of various diarrhea treatments, assess reasons for low ORS use, and identify opportunities for expanding ORS use.

**Methods:**

The project team conducted two rounds of semi–structured, quantitative surveys with more than 2000 caregivers in India and Kenya in 2012. A complementary survey covered more than 500 pharmacy staff and health care workers in both countries. In Kenya, the team also surveyed rural pharmacies to gather pricing and sales data.

**Results:**

Although caregivers generally had very positive perceptions of ORS, they typically ranked antibiotics ahead of ORS as the strongest medicine for diarrhea (in India 62% ranked antibiotics first and 23% ranked ORS first, n = 404; in Kenya results were 55% and 29%, n = 401). Many caregivers had misconceptions about the purpose and effectiveness of various treatments. For example, most caregivers who gave ORS at last episode expected it to stop their child’s diarrhea (65% in India, n = 190; 73% in Kenya, n = 154). There were noteworthy differences between India and Kenya in the selection and sourcing of treatments. Much of the money spent by families during the last episode of diarrhea was for inappropriate treatments. This was especially true in India, where rural households typically spent US$ 2.29 (median for the 79% of rural households that paid for health care services or treatments, n = 199) with most of this going to pay fees of private health workers and/or for antibiotics.

**Conclusions:**

Caregivers’ primary treatment goal is to stop diarrhea, and many believe that antibiotics or ORS will accomplish this goal. Inappropriate treatment – and especially overuse of antibiotics – is common. Satisfaction with ORS is high, but dosing is a challenge for caregivers. The results provide valuable insight into treatment behaviors and suggest significant opportunities to enhance use of ORS in developing countries.

Diarrheal disease is a leading cause of morbidity and mortality among children under five and accounts for approximately 800 000 deaths annually in this age group, mostly in developing countries [1].More than 580 million moderate to severe episodes of diarrhea occur annually in children under five, most of which result in some dehydration [2]. Since the late 1970s, oral rehydration solution (ORS) has been the most commonly recommended treatment for dehydration caused by diarrhea. More than 90% of all diarrhea deaths could potentially be avoided with universal coverage of ORS [3].

Despite the overwhelming burden of illness and the tremendous therapeutic benefits of ORS, coverage and demand in many developing countries has remained stubbornly low, with fewer than 40% of children under five in developing countries receiving ORS for the treatment of diarrhea. Coverage is particularly low among the most vulnerable: poor children and those in rural areas [4]. More specifically, in the context of ORS use or nonuse, there is a substantial “know–do gap” among caregivers. Although demographic and health survey data from many countries indicate that most mothers or caregivers are familiar with ORS as a diarrhea treatment there is little correlation between knowledge and actual use of the product [5].

The reasons for low use of ORS are a long–standing source of speculation. Some suggest that mothers do not use ORS because it does not treat the symptoms, it tastes bad, or because it does not look like a “real medicine” [6]. Others attribute low ORS use to the availability of alternative treatment products, particularly antibiotics [7]. A better understanding of the reasons for use or nonuse of ORS and other interventions could help inform public health and commercial efforts to promote or market ORS more effectively, and in turn increase coverage.

This paper presents the results of a series of surveys administered to more than 2000 caregivers and more than500 providers in India and Kenya. It examines their approaches to diarrhea treatment and the sequence of interventions used, reasons for use or nonuse of ORS and other interventions, expectations and preferences of interventions, costs associated with diarrhea treatment, and dosing. India and Kenya were selected as both are often settings of interest. While the same surveys were conducted in parallel in both countries, the study was not structured as a comparative exercise per se. These results should prove very helpful for efforts to increase ORS coverage.

## METHODS

The project team conducted two rounds of semi–structured quantitative surveys with caregivers in India and Kenya, using the same surveys and methods in both countries. It was not a comparative study and minor local variations in the surveys and methods may have occurred. The first round (R1) occurred in April and May 2012 and the second (R2) in August 2012. A complementary, semi–structured quantitative survey with pharmacy staff and health care workers was conducted in May and June 2012. Selected methods and results from the provider survey are also included in this report. In Kenya, the team also conducted a rural pharmacy survey in June and July2012 to gather pricing and sales data.

### Selection and description of participants

Quota–sampling was used to recruit two groups of caregivers: 1) those who had ever used ORS and 2) those who were aware of ORS but had never used it (hereafter referred to as ‘ever–users’ and ‘never–users’). To be eligible for either group, the caregiver must have had a child under five who had an episode of diarrhea, of any sort, within two months prior to the interview. Quotas were used to recruit approximately equal numbers of urban and rural respondents. Finally, quotas for states/provinces were used, with the sample distributed in proportion to the reported incidence of ORS usage [8,9] and spread across 18 (Kenya) to 27 (India) sampling points. States/provinces were selected to achieve appropriate representation of major socio–cultural regions, higher– and lower–coverage of ORS, areas of greater commercial relevance, and areas of more acute public health need. Within states/provinces, sampling points were purposively selected, and interviewers used systematic random sampling to identify respondents during recruitment. **Table 1** presents a profile of respondents.

**Table 1:** Characteristics of the responders in two rounds of caregiver surveys (R1 and R2)

Characteristics	India		Kenya	
	**R1 survey**	**R2 survey**	**R1 survey**	**R2 survey**
All respondents	609	404	600	401
Ever–users of ORS	320	209	320	210
Never–users, but aware of ORS	289	195	280	191
Urban	50%	50%	50%	50%
Rural	50%	50%	50%	50%
Age 18–24	34%	35%	38%	NR
Age 25–34	59%	62%	48%	NR
Age 35+	6%	4%	14%	NR
Age distribution of children 6–59 mo:*				
6–11 mo	28%	29%	31%	29%
12–23 mo	29%	27%	30%	38%
24–35 mo	30%	32%	31%	27%
36–59 mo	46%	43%	46%	47%
Hospitalized at last diarrhea episode	20 (3%)	NR	12 (2%)	NR
Socioeconomic level:†				
A, B (India, urban only; Kenya, all)	37%	35%	–	5%
C1, C2 (India, urban; Kenya, all [C1/C2])	9%	6%	48% (13/35)	57% (15/42)
D, E (India, urban; Kenya, all)	4%	9%	52%	38%
R1, R2 (India, rural)	10%	13%	–	–
R3, R4 (India, rural)	40%	37%	–	–
Region:			–	–
Uttar Pradesh (India) / Nairobi (Kenya)	124	90	97	65
Tamil Nadu (India) / Coast (Kenya)	74	43	106	71
Andhra Pradesh (India) / Nyanza (Kenya)	83	51	153	102
Maharashstra (India) / Rift Valley (Kenya)	127	93	244	163
Jharkand	31	22	–	–
West Bengal	102	69	–	–
Madya Pradesh	68	36	–	–

R1 – survey 1, R2 – survey 2, NR – not recorded

*Percentages are per age group; addition across age groups gives values over 100 percent due to the presence of multiple children in some households.

†Socioeconomic levels: A is highest and E lowest; in India, R1 denotes the highest rural grade and R4 the lowest. Socioeconomic classification was based on the standard systems used for commercial market research in the respective countries; in India, as described in “Harmonization of demographics: a manual for research agencies and users” (Market Research Society of India, Mumbai, 2011) [10]. In Kenya, the categories as initially defined by the British National Readership Survey (and often used in market research globally) were used with adapted criteria in common use among members of the Marketing and Social Research Association of Kenya.

A similar, quota–based approach was used for the survey of pharmacy staff and health care workers, and this relied on purposive sampling (Online Supplementary Document(Online Supplementary Document), table s1). Purposive selection was also used for the Kenya rural pharmacy survey, which covered 49 pharmacies in Coast, Eastern, and Nyanza provinces.

### Technical information

The R1 and R2 surveys with caregivers emphasized experience with the latest episode of diarrhea among children under five years (ie, the single most recent diarrhea episode among this age–group in the household). Topics included diarrhea duration, treatments used and sequence/timeframes of administration, and caregivers’ expectations of each treatment used – “what did you think [the treatment] would do for your child?” The R1 survey ( ~ 60 minutes) also probed for treatment source, and the R2 survey ( ~ 45 minutes) asked about treatment spending and dosing of ORS and sugar–salt solution (SSS) at the last episode of diarrhea. The spending and dosing questions merit elaboration. For each treatment they had used, caregivers were asked whether they had paid for it, and if so, about how much (except for SSS and other home remedies). Then caregivers were asked if they “had paid anything else, such as doctor’s fees, consultation fees, or clinic fees.” Interviewers recorded any items mentioned and the spending corresponding to each. The survey did not cover other costs such as for transport or lost work time. Peak dosing estimates were calculated using conservative assumptions to err on the side of over–estimating the amounts given. Caregivers were asked to think about the day on which their child’s diarrhea was “particularly bad” and were then asked how many times they gave ORS on that day and approximately how much ORS they gave each time. Frequencies of administration were recorded as ranges (eg, 1 to 2 times, 3 to 4 times) and then rounded up in the analysis (to 2 and 4 times, respectively, for example). Responses were typically expressed and recorded in terms of either “a few spoonfuls” or simple fractions of “a cup’, and high assumptions were then taken for each (eg, 25 mL for “a few spoonfuls” and 250 mL for 1 cup).

Other topics covered by the quantitative surveys with caregivers included demographics; awareness and ever–use of treatments (ORS sachets, SSS, other home remedies, antibiotics, antimotility drugs, zinc syrups/tablets, and, in India, pre–mixed ORS available in ready–to–drink packs; a localized photo–illustration card was used to assist in recall of treatment types); perceptions of ORS, using positive–negative statement pairs; and preferred speed of action and willingness to pay for a treatment for diarrhea, generally. The R1 survey also covered general attitudes and awareness in relation to treatment of diarrhea in young children, preferred product presentations, and preferred ORS pack sizes. The R2 survey included forced–rankings for the 3 main treatments (as identified at R1) on each of 4 separate parameters: effectiveness, strength, ease of use, and value.

The provider survey ( ~ 40 minutes) was tailored to the various types of health care workers and pharmacy staff interviewed. Topics included estimates of what caregivers would pay for a treatment, providers’ views of various treatments’ roles and effectiveness, profit–related influences, and caregiver expectations.

Development of the survey instruments benefited from a careful process of formative research and pretesting. Formative research was conducted with all respondent types in multiple regions of each country. Open–ended, in–depth individual interviews and group discussions were used to explore the range of attitudes and behaviors, including perceptions of treatments and possible product features. In both countries, a small team of 2–3 experienced local researchers led all of the interviews/discussions and performed the analysis. These researchers and the core team met in a workshop to bridge from the formative research to design of the surveys and instruments. The R1 survey instrument went through two rounds of pretesting with a few dozen respondents in each country. Special attention was given to the questions relating to treatment behaviors at last episode. The R2 instrument was lightly pretested; many of its main questions were carried over from the R1 survey, initial results of which were utilized to inform design of the R2 instrument.

### Statistical methods

The closed–ended data from both the R1 and R2 surveys were analyzed in a similar manner. The initial, base analysis included an examination of data at a total respondent level as well as by key groups such as ever–users vs never–users, urban vs rural. Additional subgroups of interest were identified during the analysis to elicit areas of differentiation or drivers of behavior. An extensive series of subgroup analyses were performed. For example, the possibility of differences in treatment behaviors between various socioeconomic and demographic subgroups was carefully explored. All salient findings are presented as results. Certain forms of questioning also called for specific types of analyses. For example, because a broad set of detailed questions was asked about the last diarrhea episode, subsequent analysis required aggregation across the episode at an individual respondent level to build patterns of product usage. Although elements of the initial base analysis were designed a priori, much of the analysis was conducted post hoc after the complete results of each survey were ready for analysis.

Open–ended data from the R1 and R2 surveys were analyzed through a similar procedure. The process began with review of verbatim responses for each question. Key common themes were identified for each question, as well as factors associated with each theme. This represented a code frame. Each verbatim response was then analyzed and assigned to its appropriate code.

## RESULTS

### Caregiver perceptions and expectations influencing treatment decisions

When caregivers were asked about their expectations for treatment, “Stop the diarrhea” was the most frequent response (**Table 2**). When they were asked “What is the longest period of time that would be acceptable to you from first giving your child something for diarrhea until the child’s bowel movements return to normal?”, the mean response (±standard deviation) was 1.7 ± 1.2 days in India and 1.6 ± 1.1 in Kenya (Online Supplementary Document(Online Supplementary Document), table s2).

**Table 2:** Expectations (% respondents) of main treatments, when used to treat at last episode of diarrhea

Principal expectations of treatments (%)	India			Kenya		
	**Antibiotics (n = 238)**	**ORS (n = 190)**	**SSS (n = 270)**	**Antibiotics (n = 189)**	**ORS (n = 154)**	**SSS (n = 155)**
Stop the diarrhea	91	65	41	70	73	59
Replace fluids lost due to diarrhea	11	62	73	13	51	39
Improve child’s energy level	43	59	31	17	36	14
Improve child’s health	51	38	32	8	8	6
Reduce frequency of bowel movements	37	33	26	10	8	14
Reduce vomiting or fever†	28	23	24	–	–	–
Help treat diarrhea faster	24	15	6	12	5	6
Reduce pains†	–	–	–	18	6	15
Kills all bacteria or germs†	–	–	–	32	5	7

ORS – oral rehydration solution, SSS - sugar–salt solution

*R2 survey. This exercise was used in open–ended format in the R1 survey, and in closed–ended format at R2. Analysis of R1 results led to the pre–codes used at R2; the option of an open–ended ‘other’ response was included, and respondents were not prompted at R1 or R2.

†Indicates expectations which were not mentioned for any main treatment by at least 8% in one of the countries (India or Kenya), and have been truncated with “–“.

**Table 3** shows how caregivers ranked treatments based on 4 criteria: most effective at treating the cause of diarrhea, strongest medicine for diarrhea, easiest to get children to take, and best use of money to treat diarrhea. Antibiotics tended to outrank ORS in both countries, although ORS ranked appreciably better in Kenya than in India. More caregivers ranked antibiotics first on effectiveness and strength than ranked ORS first on these attributes, although the margin in favor of antibiotics was narrower in Kenya. Kenyan caregivers were split equally as to whether antibiotics or ORS represent the best use of money, but Indian caregivers favored antibiotics over ORS by 3 to 1 on value. Among caregivers who used both antibiotics and ORS for the last episode, the differences in results between India and Kenya are even more pronounced (**Table 3**). In Kenya, most ranked ORS ahead of antibiotics on both effectiveness and value, while ranking antibiotics ahead on strength by a margin of 3 to 2. In India, by contrast, antibiotics were ranked much further ahead of ORS on these attributes.

**Table 3:** Ranking of main treatments (% rating as the 1st choice)

Treatment	All respondents		Used antibiotics and ORS both*		Used antibiotics but not ORS*		Used ORS but not antibiotics*	
	**India (n = 404)**	**Kenya (n = 401)**	**India (n = 131)**	**Kenya (n = 79)**	**India (n = 112)**	**Kenya (n = 154)**	**India (n = 58)**	**Kenya (n = 58)**
Most effective at treating the cause of diarrhea:								
Antibiotics	52	45	59	46	86	48	9	22
ORS	25	35	37	53	–	14	78	74
SSS	23	20	5	1	14	39	14	3
Strongest medicine for diarrhea:								
Antibiotics	62	55	67	58	88	57	12	34
ORS	23	29	31	41	–	10	78	62
SSS	15	15	2	1	12	32	10	3
Easiest to get children to take:								
Antibiotics	22	35	15	28	44	40	3	17
ORS	31	39	60	61	–	10	67	81
SSS	47	26	2	11	56	49	29	2
Best use of money to treat diarrhea:								
Antibiotics	73	50	63	41	100	73	14	24
ORS	27	50	37	59	–	28	86	76

ORS – oral rehydration solution, SSS - sugar–salt solution

*Used at last episode of diarrhea.

Among caregivers who used antibiotics and *not* ORS during the last episode, a group that could be expected to most favor antibiotics, results from India were heavily in favor of antibiotics. The same group in Kenya, however, was less enthusiastic about antibiotics and ranked them similarly on effectiveness and strength as did Kenyan caregivers who had used both antibiotics and ORS. Also, many ranked SSS first (**Table 3**).

When caregivers were asked about their expectations for specific treatments during the most recent episode ‘Stop the diarrhea’ was the most common expectation for both antibiotics and ORS in both countries (**Table 2**). Antibiotics scored much higher than ORS on “stop the diarrhea” in India (91% vs. 65% across all users; and 95% vs. 53% in the two lower, rural socioeconomic grades), and they scored on par with ORS in Kenya (70% vs.73%, **Table 2**). Expectations results for the two lower, rural socioeconomic grades in India correspond to grades R3 and R4 combined (antibiotics, n = 238; ORS, n = 190; SSS, n = 270). Caregivers’ expectations for treatments were broadly consistent with the reasons they gave for their ranking of the main treatments (Online Supplementary Document(Online Supplementary Document), table s3).

Although ORS outscored SSS on most benefits in both countries, SSS did score higher on “replace fluids” response in India, where caregivers in the two lower rural socioeconomic grades had especially high expectations for ORS (73%) and SSS (84%) to replace fluids. In Kenya, fluid replacement was mentioned less often for both ORS (51%) and SSS (39%) as compared to India (62% and 73%, respectively; **Table 2**). More than half of health care workers and pharmacy staff in Kenya and more than one–third in India agreed with the statement “mothers do not really understand that ORS rehydrates the child” (Online Supplementary Document(Online Supplementary Document), table s4).

**Table 4** focuses on caregiver perceptions of ORS, as reflected in agreement or disagreement with various statements about ORS. Overall, perceptions of ORS were quite positive among both ever– and never–users. Most caregivers in both countries indicated that they believed ORS is a medicine and that it stops diarrhea. This was true for both ever– and never–users.

**Table 4:** Caregiver perceptions (% response) of ORS, on Positive [negative] paired statements*

Perception	India				Kenya			
	**Ever–users of ORS (n = 320)**		**Never–users but aware of ORS (n = 289)**		**Ever–users of ORS (n = 320)**		**Never–users but aware of ORS (n = 280)**	
	**Positive**	**Negative**	**Positive**	**Negative**	**Positive**	**Negative**	**Positive**	**Negative**
Helps [does not help] replace fluid/water and minerals lost due to diarrhea	98	1	91	2	96	2	74	5
Easy [difficult] to obtain such products	97	2	91	4	88	11	74	12
Restores [does not] the child’s energy and appetite	97	2	85	3	89	6	62	9
ORS increases [does not] the child’s energy	97	1	87	1	90	7	61	8
Instructions on how to prepare it are clear [unclear]	97	1	86	4	95	3	64	6
I am confident [not confident] that the water I use to make ORS is clean	94	4	90	5	90	7	57	18
Rehydrates [does not rehydrate] the child	94	2	91	2	96	3	76	4
Easy [difficult] to prepare	92	6	91	2	94	5	75	8
Reduces [does not reduce] the child’s bowel movements	92	4	82	6	85	12	60	16
I feel [do not feel] confident that I know how to prepare ORS properly	92	7	83	6	93	6	54	24
Stops the diarrhea [does not stop the diarrhea]	92	6	79	4	80	17	57	18
Relieves [does not relieve] stomach pains the child may have	91	2	76	3	67	19	41	18
Does not take a lot of time and effort to prepare [takes a lot of time and effort]	88	11	83	13	89	10	64	13
Easy [difficult] to get the child to drink it	88	11	68	16	66	32	42	30
Does not usually [usually] cause children to vomit	84	10	59	15	71	25	46	18
Not an expensive treatment [expensive treatment]	83	14	78	16	88	7	74	8
Easy [difficult] to obtain clean water to make it	83	16	71	26	75	22	68	21
Is [not] a medicine	78	20	68	27	92	6	77	9
Nice pleasant taste [not nice and unpleasant taste]	75	17	66	10	57	38	31	35
Rarely [often] have liquid left over which is wasted	60	36	47	31	54	42	31	32
The frequency of giving these products to the child is acceptable [you need to give these products to the child too often]	38	59	20	64	65	32	49	23
Not too much liquid for a young child to take [too much liquid for a child to take]	15	82	14	73	67	30	45	25

ORS – oral rehydration solution

*Positive – chose ORS–positive statement; Negative– chose ORS–negative statement. “Don’t know” responses are not shown, but are equal to 100% less the sum of the positive and negative response percentages; DK responses were more common among never–users.

There was concern among caregivers in both countries, however, that ORS was too much liquid for a child to take, that it needs to be given too often, and that there is often liquid leftover that goes to waste. The first two of these concerns were mentioned more frequently in India (82% and 73%) than in Kenya (30% and 25%). In Kenya, about one–third of caregivers indicated dissatisfaction with the taste of ORS and the difficulty of getting a child to drink it. These issues were not as important among caregivers in India (**Table 4**). In R1 interviews, 97% of caregivers (n = 609) in India and 92% (n = 600) in Kenya agreed with the statement that “children with diarrhea need more water and liquids”.

### Treatment choice, timing, dosing, and sourcing

Antibiotics were used to treat about half of episodes in both countries, regardless of ORS use (**Table 5**). Most ORS ever–users also reported use of ORS during the last episode (India 91%, Kenya 75%). Use of SSS or other home remedies was fairly common among both ORS ever–users and never–users and was higher in India than in Kenya. Reported use of other treatments, such as zinc and antimotility drugs, was low.

**Table 5:** Treatments given at last episode of diarrhea*

Treatment	India		Kenya	
	**Ever–users of ORS**	**Never–users but aware of ORS**	**Ever–users of ORS**	**Never–users but aware of ORS**
All treatments given, as % (n = episodes treated):	n = 524	n = 464	n = 494	n = 363
Antibiotics	59	54	48	56
ORS	91	–	75	–
SSS	58	62	31	44
Other home remedy	27	31	9	14
Antimotilities	3	3	10	11
Zinc	12	8	9	6
Monotherapy only, as % of episodes treated	6	24	36	65
Monotherapy only (n = episodes so treated)	n = 49	n = 196	n = 178	n = 238
Antibiotics as % of monotherapies	16	39	23	47
ORS as % of monotherapies	58	–	52	–
SSS as % of monotherapies	16	42	14	30

ORS – oral rehydration solution, SSS - sugar–salt solution

*Combined results from R1 and R2 surveys, weighted equally.

Use of only one treatment (ie, monotherapy) was more common in Kenya than in India (**Table 5**). In Kenya, 36% of ORS ever–users and 65% of never–users used monotherapy (most often antibiotics). In India, by contrast, only 6% of ORS ever–users and 24% of never–users used monotherapy, reflecting higher reported use of ORS, SSS, and other home remedies. In India there were also notable differences in treatment usage between socioeconomic grades, with more antibiotic use among lower, rural grades and greater use of SSS and other home remedies among higher grades (Online Supplementary Document(Online Supplementary Document), table s5).

The duration of the last episode was typically 3 to 4 days in both countries (Online Supplementary Document(Online Supplementary Document), table s6). When given, SSS or other home remedies were typically started on day 1 and given for a median of 3 days in India and 2 days in Kenya (Online Supplementary Document(Online Supplementary Document), table s6). When ORS was given, it was typically started on day 2 and given for 3 days. When antibiotics were used, they were typically started on day 2 and given for 3 days in India or 4 days in Kenya. Most Indian caregivers (75%) took action on day 1 by adjusting the child’s diet or starting a treatment. Forty percent of Kenyan caregivers, however, took a wait–and–see approach, typically for one full day.

Caregivers who gave ORS typically used a relatively low amount on the day of peak use and believed it was the most they could give their child in one day. In both countries, two thirds of those using ORS with children 6 to 24 months old (primarily 12 to 24 months old) gave less than 500 mL per day at peak, typically giving 250 mL in Kenya and 125 mL in India (median values; **Table 6**). Among caregivers in both countries who used ORS with children 24 to 60 months old, the majority gave less than 1 L per day at peak. In Kenya, these caregivers typically gave 250 mL, and in India 375 mL (medians). When SSS was used, peak dosing levels generally followed the same pattern as for ORS. Among caregivers in both countries who used ORS, at least 73% said the amount they gave per day at peak was the maximum amount they could give their child each day. Notably, caregivers in both India and Kenya who reported giving more ORS than the thresholds applied at the analysis stage (500 mL or 1 L, per age group) were also more likely to indicate it was the maximum amount they could give their child on one day (**Table 6**).

**Table 6:** Dosing at last episode of diarrhea, ORS and SSS

Dosing	India				Kenya			
ORS:*								
Age treated at last diarrhea episode: range*	6 mo to <2 y (n = 61)		2–5 y (n = 96)		6 mo to <2 y (n = 42)		2–5 y (n = 56)	
ORS given on day of peak use (mL)	<500	≥500	<1000	≥1000	<500	≥500	<1000	≥1000
Percentage	67%	33%	82%	18%	64%	36%	91%	9%
Median (IQR) amount ORS given, day of peak use	125 mL (150)	750 mL (250)	375 mL (375)	1125 mL (500)	250 mL (150)	500 mL (375)	250 mL (275)	1000 mL (500)
Caregivers who felt this was the maximum amount they would be able to give their child in one day	85%	95%	73%	100%	78%	87%	75%	80%
SSS:*								
Age treated at last diarrhea episode: range	6 mo. to <2 y (n = 78)		2–5 y (n = 135)		6 mo. to <2 y (n = 48)		2–5 y (n = 56)	
SSS given on day of peak use (mL)	<500	≥500	<1000	≥1000	<500	≥500	<1000	≥1000
Percentage	78%	22%	95%	5%	73%	27%	98%	2%
Median (IQR) amount SSS given (mL), day of peak use	250 (125)	500 (0)	375 (250)	1000 (0)	125 (150)	625 (375)	125 (150)	1000 (0)

ORS – oral rehydration solution, SSS – sugar–salt solution, mo – month, y – year

*R2 survey. Regarding the n values in this table: For the dosing analysis, only data from households with one child aged 6–59 mo were used, to allow for sub–analysis by the two age groups shown; although households with more than one child aged 6–59 mo were also included in the surveys, and the number and ages of all such children was recorded per household (reflected in the age distribution data presented in Table 1), record was not made of which specific child had suffered the most recent episode of diarrhea. *In this range, most were aged 1 year to <2 years.

In India, private facilities were the primary source of both ORS (80%) and antibiotics (80%) (Online Supplementary Document(Online Supplementary Document), table s7). In Kenya, by contrast, public facilities clearly played a much larger role as sources of ORS (67%) and antibiotics (48%), though a quarter of responses about sources of antibiotics in Kenya could not be categorized.

Most caregivers in both countries who used ORS or antibiotics reported doing so on a provider’s recommendation. In India, recommendations figured more prominently with use of antibiotics (95%) than with use of ORS (75%). In Kenya, the situation was reversed: ORS (89%) was more likely to be used on recommendation than antibiotics (77%). The data thus suggest that nearly a quarter of caregivers in Kenya used antibiotics without a recommendation from a health care provider or pharmacy worker (Online Supplementary Document(Online Supplementary Document), table s7).

Among health care workers and pharmacy staff in both countries, 82% or more agreed with the statement “mothers like to be given the most powerful treatments”. Except for health care workers in Kenya, most also agreed that “antibiotics are the most effective treatment for diarrhea” (Online Supplementary Document(Online Supplementary Document), table s5). In rural areas of Kenya, pharmacy staff estimated that caregivers walk 7 km one–way, on average, to reach their pharmacy (n = 49), and they estimated that one half of their clients are mothers of young children under five years old (n = 34).

### Financial considerations and preferred product formats

In India, median spending on the last episode of diarrhea was US$ 2.70 (**Table 7**). Among rural respondents, many of whom were at the lowest socioeconomic levels, it was US$ 2.29. The largest share of spending went to ‘doctors’ for services. About 72% of caregivers paid for these services, spending a median US$ 1.80 (US$ 1.26 in rural areas). These services may have included fees for injections or administration of intravenous fluids, which typically account for a significant portion of spending in this category.

**Table 7:** Caregiver spending at last diarrhea episode and related profitability for rural private providers*

Parameter	India		Kenya	
	**All**	**Rural**	**All**	**Rural**
Carers who paid for health care services/other *and/or* treatments	79% (n = 397)	79% (n = 199)	64% (n = 352)	63% (n = 168)
Total spent, among all carers who paid (median) (IQR)	US$ 2.70 (2.9) (n = 313)	US$ 2.29 (1.96) (n = 158)	US$ 0.82 (1.4) (n = 225)	US$ 0.70 (0.82) (n = 105)
Treatments, spending – main components:				
Antibiotics – of carers who gave at last episode,% who paid	93% (n = 238)	94% (n = 116)	73% (n = 189)	62% (n = 93)
Antibiotics – median amount spent at last episode (IQR) by carers who paid	US$ 0.90 (1.03) (n = 221)	US$ 0.90 (0.68) (n = 109)	US$ 0.59 (0.59) (n = 138)	US$ 0.59 (0.35) (n = 58)
Antibiotics – gross profit^a^ (gross margin^b^) at retail–level, rural		US$ 0.23 (25%)^c^		US$ 0.24 (41%)^d^
ORS – of carers who gave at last episode, % who paid	73% (n = 190)	71% (n = 97)	43% (n = 156)	29% (n = 77)
ORS – median (IQR) amount spent at last episode, among carers who paid	US$ 0.27 (0.25) (n = 139)	US$ 0.27 (0.20) (n = 69)	US$ 0.47 (0.47) (n = 67)	US$ 0.59 (0.35) (n = 22)
ORS – gross profit^a^ (gross margin^b^) at retail–level, rural		US$ 0.09 (33%)^c^		US$ 0.24 (41%)^d^
Healthcare services/other, spending: main components				
India – “Doctors fees”^f^: carers who paid fees at last episode, as% of all	72% (n = 404)	74% (n = 202)		
India – “Doctors fees”^f^: median (IQR) spending at last episode	US$ 1.80 (2.7) (n = 290)	US$ 1.26 (0.9) (n = 149)		
India – “Doctors fees”^f^: profit^a^ (gross margin^b^) at retail–level, rural		US$ 0.58 (46%)^e^		
Kenya – median (IQR) “card registration fees” ^g^ (occasional public–sector user fee)			US$ 0.35 (0.35) (n = 36)	US$ 0.35 (0.35) (n = 26)
Profitability analysis, retail–level: typical carer purchases, rural				
Total gross profit^h^ – when carer purchases all of above^i^ (illustrative)		US$ 0.90		US$ 0.48
Share of total gross profit from ORS, in this scenario:		10%		50%

ORS – oral rehydration solution

*R2 survey except where otherwise noted. See also Methods for how caregivers’ spending was recorded. Notes: ^a^Calculated as median amount spent x gross margin; ^b^Gross margin taken here as equal to [(Revenue) – (Cost of materials)]/(Revenue) and excludes cost of labor, transport, rent, etc; ^c^India – estimate from discussion with pharmaceutical executives by PATH, n = 3; ^d^Kenya rural pharmacy survey, n = 49; ^e^Radwan [14], 2005; ^f^The “doctors fees” category was prominent in India results and included fees for injections or administration of intravenous fluids, which typically account for a significant portion of spending with private practitioners in that country; ^g^This category is specific to Kenya results, and represents the co–pay sometimes charged by public clinics, which then provide a consultation and treatments (e.g., ORS), if in stock, for no added cost; ^h^Gross profit accruing to private pharmacy in Kenya, and in India accruing directly to the private health–care worker and their affiliated pharmacy (in India, 65% of private health–care workers stated caregivers would obtain the products they prescribed either from their own practice (44%) or an affiliated pharmacy (21%; n = 63)); ^i^Sum of gross profits for antibiotics, ORS, and (in India) doctors’ fees. Amounts shown in US dollars (US$), based on current exchange rates: India, 55.4 INR = US$ 1; Kenya, 84.7 KSH = US$ 1.

In India, the cost of antibiotics was the other main component of spending. Almost all caregivers (93%) who used antibiotics during the last episode paid for them. Median spending was US$ 0.90, both for all users and for rural users. Although most caregivers who used ORS paid for it (73%), median spending on this product was only US$ 0.27, both for all users and rural users. Spending of US$ 0.27 matches the observed rural price of a single 1L ORS sachet, the size typically used in India.

In Kenya, median spending on the last diarrhea episode was US$ 0.82 (US$ 0.70 in rural areas) (**Table 7**). The largest amount of spending was for antibiotics. Most caregivers (73%) who used antibiotics paid for them. Median spending for antibiotics was US$ 0.59 both for all users and rural users and was consistent with purchase of syrup presentations. Syrups accounted for 100% of rural pharmacies’ fastest–selling antibiotics for diarrhea, with a median price of US$ 0.59 (rural pharmacy survey; n = 125 products and prices, n = 49 pharmacies). The consistency between caregivers’ self–reported spending on antibiotics at last episode and the product and pricing data gathered from staff at rural pharmacies is noteworthy.

Almost half (43%) of Kenyan caregivers who used ORS reported paying for it. Median spending was US$ 0.47 for all users and was actually higher among rural users at US$ 0.59 (**Table 7**). These figures imply purchase of 2.6 and 3.3 sachets, respectively, at the prevailing unit price of US$ 0.18 for one 0.5 L ORS sachet (the typical size in Kenya; price from rural pharmacy survey, n = 49). The numbers of sachets purchased approach the quantity Kenyan public sector health care workers said they prescribe (median 4, n = 90).

Selling ORS in rural areas appears to carry more profit potential at the retail level in Kenya than in India (**Table 7**). In Kenya, the retailers’ profit incentive is to sell either antibiotics or ORS – and preferably both – since each will provide equal gross profit of US$ 0.24. In India, the retailers’ incentives are to focus first on selling services (eg, doctors’ fees, which may often include injections) and antibiotics, whereas selling ORS offers only a minor boost to gross profit. It is important to remember that the treatment components (antibiotics, ORS, etc.) were used at last episode to varying degrees and purchased privately to different degrees, so the profit breakdown and totals shown in **Table 7** are only illustrative.

Caregivers’ stated willingness to pay for a full course of “a treatment for diarrhea, considering you might need to use the product for 5 days” was consistent with their purchase behavior and estimates by health care workers and retailers. In India, caregivers’ (n = 404) stated willingness to pay was a median of US$ 1.80 (interquartile range (IQR) = 2.8) vs spending on the last episode of US$ 2.70 (IQR = 2.9). Estimates of what caregivers would be willing to pay by health care workers (median US$ 1.80, IQR = 2.7, n = 134) and pharmacy staff (median US$ 1.80, IQR = 1.8, n = 121) were similar, and aligned to caregiver estimates.

In Kenya, caregivers (n = 401) said they would be willing to pay a median of US$ 0.60 (IQR = 0.82) vs spending on the last episode of US$ 0.82. Estimates by pharmacy staff (US$ 1.20, IQR = 1.8, n = 144) were higher than caregiver estimates while health care workers (median US$ 0.60, IQR = 1.2, n = 117) were more aligned with caregiver estimates.

When asked about preferred product formats, caregivers generally expressed a preference for a conventional ORS sachet or either of two ready–to–drink formats (Online Supplementary Document(Online Supplementary Document), table s8). There was a stronger preference for the conventional ORS–style sachet in India (41%) than in Kenya (21%), and the combined preference for the two ready–to–drink formats was similar in the two countries (42% and 49%, respectively). There was modest interest in a syrup presentation and minimal interest in either a self–dispersing tablet presentation or an alternative sachet style.

## DISCUSSION

### Caregivers’ primary treatment goal is to stop diarrhea

Caregivers give treatments they believe will treat the diarrhea. Strikingly, the most frequent expectation of either antibiotics or ORS was that these treatments would stop the diarrhea. This motivation accounts for a substantial proportion of the actual use of ORS even though the real benefits of ORS center on rehydration. Although this finding contrasts with the results of some other surveys [11], caregivers’ emphasis on treating the diarrhea has often been reported [12]. The use of multiple treatments by many caregivers – especially common in India – may derive in part from caregivers’ perceptions that several of the main treatments are efficacious against the diarrhea itself. To state it simply, while many caregivers give antibiotics primarily to treat the diarrhea itself and very few perceive a rehydration benefit with antibiotics, their use of ORS or SSS may be driven as much by perceived efficacy against diarrhea as by rehydration goals. The widespread use of antibiotics is consistent with the alignment between caregivers’ emphasis on treatment and their perception that antibiotics are the most effective and strongest medicine for diarrhea.

Several issues related to the timing, sequence, and the number of treatments given at last episode are worth highlighting. Although many caregivers in Kenya gave only one treatment, many in India deployed the proverbial ‘kitchen sink’ of treatments. Each of these patterns may provide distinct challenges for product promotion initiatives. For example, when caregivers are accustomed to giving one treatment, it may be hard to displace incumbent treatments or to catalyze demand for consistent use of both ORS and zinc (instead of one or the other).

If home remedies were used, they were typically started on the first day of the episode, and products such as antibiotics and ORS were typically started on the second day and continued for 3 days (4 days for antibiotics in Kenya). Because most episodes of diarrhea are self–limiting and typically last only 3 to 4 days, caregivers may form incorrect perceptions of the efficacy of some treatments. That is, caregivers may assume that the diarrhea stopped because of treatments being given at the time. There are several implications for intervention design. For example, promoting rapid initiation of ORS may be counterproductive to caregiver demand for ORS unless equal emphasis is placed on sustaining use through the episode. Also, promoting the benefits of ORS may be counterproductive if this focuses too narrowly on the rehydration benefit and so creates dissonance with the perception of many existing ORS users that the product helps treat diarrhea (possibly eroding use or users’ potential to function as effective product advocates).

Although caregivers’ primary focus is on treatment of diarrhea, the results of our interviews show that almost all caregivers understand that children with diarrhea need more fluid and that ORS helps replace fluid and minerals lost to diarrhea. This is important in part because some public health advocates may assume this is not understood, as did many of the providers surveyed.

### Inappropriate treatments are strongly entrenched

Use of inappropriate treatments was widespread, consumed most of caregivers’ spending on treatment, and represents the expected standard–of–care from the viewpoints of both providers and caregivers. These widely documented [11,13,14], and well–established behaviors constrain the potential for correct and consistent use of ORS (and zinc) by diverting caregivers’ limited time, money, and energy.

Indian caregivers’ high spending was noteworthy. They obtained most treatments and health care services from private sources at last episode. The largest component of spending was for payments to private practitioners. We suspect a substantial share of this spending likely went for injections and intravenous fluids administration, based on reports of practices typical in India, consistency with the spending levels we observed, and private–practitioners’ tendency to charge principally for injections and medicine rather than the consultation itself (Anna Stratis, personal communication, 2012) [15]. For example, Bhatia reported injections were given in two–thirds of 451 private–sector consultations [16], and Ashwath reported half of 64 children attending an outpatient–hospital clinic received an injection for an illness within the previous month [17].

The higher spending per episode in India may help to explain why Indian caregivers typically started treatment more quickly and used more treatments (including more homemade treatments such as SSS, very early in the episode) than their counterparts in Kenya. That is, Indian caregivers’ may be trying more energetically to head off the diarrhea episode before it progresses and occasions what they know to be a costly set of treatments.

In Kenya, outlays per episode were still substantial relative to caregivers’ limited incomes, although these costs are clearly much lower than they would be absent the subsidized care and treatments from the public sector. Our results illustrate one potential benefit of the relatively more involved public sector as seen in Kenya, and that is the norming effect such public services can have when they are provided widely. When Kenyan caregivers did need to purchase ORS privately, they typically purchased about 3 sachets of 0.5 L, approaching the number of sachets they would typically receive from public sources and spending a substantial US$ 0.47 to US$ 0.59 to do so.

A corollary observation is that the antibiotics used were typically not much more expensive than a course of ORS in the private sector, even before considering the time–and–effort costs of administering ORS or availability of antibiotic tablets that often cost less than a sachet of ORS [12,13,18]. This is important in part because some public health advocates may assume ORS is much less costly than antibiotics.

The results suggest caregivers, health care workers, and pharmacy retailers are conditioned to the now–routine use of antibiotics with pediatric diarrhea. Caregivers rank antibiotics ahead of ORS as the most effective treatment against the cause of diarrhea and as the strongest medicine. Caregivers may play an important role influencing providers’ treatment recommendations. After all, and as illustrated in rural Kenya, mothers of young children form a large part of pharmacies’ ‘customer base’ and often must walk many kilometers to visit a pharmacy (ie, they may not be inclined to go home ‘empty handed’ or with ORS only). Most providers felt their clients want to receive the most powerful medicines, and (like caregivers) most of them felt antibiotics are the most effective medicine for diarrhea in young children. Although most Kenyan health care workers disagreed that antibiotics are the most effective treatment, approximately half of the antibiotics used by Kenyan caregivers at last episode were obtained from public facilities like those where most of the health care workers interviewed in Kenya were employed. The difficulties experienced by provider–training initiatives on treatment of pediatric diarrhea in developing countries [19] are consistent with our conclusions. Profitability also plays a role but to a lesser extent than is often imagined; for example, in Kenya the public sector provided about half of the antibiotics used to treat recent episodes of pediatric diarrhea.

The emergence of antibiotic (over)use as an established norm, and the role of client expectations, is not isolated to developing countries or to diarrhea. An example from the United States offers striking parallels. In the United States, sinusitis is the fifth leading driver for antibiotic prescriptions even though about 80% of cases will cure without medication and viral infections cause up to 98% of sinusitis. However, “antibiotics are prescribed more often than not, which reflects patients’ expectations and the problem of differentiating viral from bacterial sinusitis in the primary care setting” [20].

The common use of antibiotics raises health and safety concerns, as do indications (in India) of widespread use of injections. Our results are consistent with reports on the overuse of antibiotics and contributing factors [21], both with diarrhea and generally [22-24]. Taken together, these raise important concerns about the potential development of antibiotic resistance [25,26]. In Kenya, the frequent use of antibiotics without any rehydrating therapy is worrisome. If zinc were to displace antibiotics, it might be administered without ORS or SSS. Finally, the possible widespread use of injections by private practitioners in India raises obvious safety concerns, emphasizing the potential for tragedies similar to those described in press reports [27].

### Satisfaction with ORS is high, but dosing is a challenge

An encouraging finding is the strong pattern of re–use of ORS by caregivers, who seem substantially satisfied with the product. If a caregiver had any previous experience using ORS, she typically obtained and used ORS during the last episode. This suggests that ORS product innovations such as taste improvements or packaging enhancements may offer less scope for improvement in ORS coverage than facilitating (first) use experiences and access among target populations.

Strikingly, both ever– and never–users of ORS held quite positive perceptions of ORS. This is consistent with the high rate of use of ORS at last episode among caregivers who had ever used ORS. The few concerns expressed mostly related to the volume of liquid, the (related) need to administer ORS too frequently, and often having leftover liquid that is wasted.

When caregivers did use ORS, most gave a relatively small volume of the solution each day at the peak of the episode, and most felt it was all they could manage to administer. Concerns about the volume of liquid and the frequency of administration were prominent among caregivers in both countries. Simply put, caregivers feel that the recommended dosing of ORS is impractical and overly ambitious, and they are giving less. Our results were consistent with previous reports on under–dosing of ORS [28]. The disconnect between the recommended dosing of ORS (geared towards particularly acute diarrhea, such as with cholera) and what is practical for caregivers dealing with more typical diarrhea may challenge caregivers’ feelings of self–efficacy, creating disincentives to rapid initiation of ORS and correct use. Research to investigate a more practical yet sufficiently effective dosing regimen may be warranted. Because our results raise doubt on correct use, they call into question reliance on the simple metric of “ORS coverage”, which only addresses consistency of use.

Preparation of the solution is another important aspect of correct use. Although we did not study this aspect of dosing directly, the data on low dosing of liquid, duration of use (3 days), and the number of sachets actually purchased (in India, only one) together suggest that many caregivers may be using ORS “a pinch at a time” and thus in highly variable concentrations. Other reports have indicated that problems with correct preparation of ORS are common [29,30].

Most caregivers in both countries preferred a 200 mL ORS pack size over a 1 L size (currently, typical pack sizes are 500 mL in Kenya) and 1 L in India). The smaller pack size would help to address caregivers’ concerns on wastage of leftover ORS. While many were attracted to ready–to–drink formats, the higher cost would likely limit uptake (this is the case in India, where ready–to–drink ORS is sold; reported use was low).

ORS is perceived as offering superior benefits compared to SSS, generally. ORS is perceived to offer the most balanced mix of expected benefits. It also ranked ahead of SSS on effectiveness and strength. Caregivers appear somewhat split on whether ORS or SSS is more acceptable to children, with a lean towards SSS in India and towards ORS in Kenya. A substantial minority of Kenyan caregivers voiced concern on the taste of ORS but a majority ranked ORS first on acceptability to children, with many citing good taste. Strong majorities of Indian caregivers felt ORS has a pleasant taste, but more ranked SSS first for acceptability to children than did so for ORS. In both countries, partisans of ORS or SSS all typically cited good taste in their rationale. If there were a major underlying difference in acceptability, we would expect to see this reflected in dosing, but the pattern of dosing amounts for ORS are broadly in line with dosing of SSS. If SSS had an advantage, we may also expect lower re–use of ORS among ever–users of ORS, but this is not what we observed. Experience and habits may play a powerful role in sustaining use of either ORS or SSS. Affordability and accessibility may be the primary advantages of SSS, so enhancing affordability and accessibility of ORS could be important to displace SSS use. However, it is not immediately clear what priority displacing SSS with ORS merits given the scale of problems such as antibiotic overuse, antibiotic use in the absence of any rehydrating therapy (in Kenya), or the potentially widespread use of unnecessary injections (in India).

### Limitations

The study has some noteworthy limitations. First, the surveys were non representative because we used quota–sampling methods and excluded caregivers who were not aware of ORS. However, ORS awareness is high, we had many respondents from diverse settings and socioeconomic strata, and the results are highly consistent. Second, resource limitations prevented a second–round survey of providers. Third, we did not attempt to record co–morbidities or severity of diarrhea at last episode because of concerns about reliability and the expected low incidence of severe cases. Fourth, we did not measure response rates. Fifth, a caregiver’s recall of a diarrhea episode that occurred up to two months ago may be inaccurate. However, it is expected that many individual–level inaccuracies cancel out with respect to the summary statistics reported, and every effort was made to avoid introducing systematic biases. Also, recall–based results were remarkably consistent with other results. These considerations mitigate concerns about the reliability of information based on recall.

While this study was implemented in both India and Kenya, it was not designed as a comparative exercise per se. The reader should use caution in interpreting any apparent national–level differences.

Other factors also need to be considered when interpreting the results. For example, some caregivers may not have known what medicines they gave—a particular concern in differentiating antibiotics from antimotility drugs. However, the reported durations of antibiotic administration (median 3 days in India, 4 days in Kenya) and of diarrhea episodes (continuing, on average, for 3 days after initiation of antibiotics) are inconsistent with what would be expected had modern antimotility drugs (eg, loperamide) been given. Further, the amounts spent on antibiotics were consistent with purchase of syrup presentations, which are often preferred and are sold in single–course bottles that are more readily distinguishable than tablets. Extensive pretesting found respondents easily chose treatment categories from the visual cards used. When interviewers presented the visual cards for each category they also mentioned some common drugs as examples, such as the Flagyl and Norflox/Oflox trade–name antibiotics often used with diarrhea in Kenya and India, respectively. While all of these considerations mitigate concern on reliability of recall of drugs, this remains a limitation given the variety of drugs and variable knowledge of caregivers.

In India, we decided not to probe into the use of injections or intravenous drips, given concern that the complexities of studying these practices would spread the survey too thin. However, the high spending on “doctors fees” recorded in round 2 was a surprise and may indicate widespread use of injections and intravenous drips. This deserves further research.
