# Supplementary material for: Treatment of diarrhea in young children: results from surveys on the perception and use of oral rehydration solutions, antibiotics, and other therapies in India and Kenya
Source: J Glob Health. 2013 Jun;3(1):010403. doi: 10.7189/jogh.03.010403 (PMC3700033; doi:10.7189/jogh.03.010403)
Supplement: Online Supplementary Document [file jogh-03-010403-s001.pdf]

Online Supplementary Document

Zwisler et al. Treatment of diarrhea in young children: results from surveys on the perception and use of ORS, antibiotics, and other therapies in India and Kenya.

Journal of Global Health 2013;3:010403

**Table s1: respondents, health care workers and pharmacy staff**			
**INDIA**	**Total**	Rural	Urban
Health care workers, total (public and private)	134	73	61
Public:			
Clinical officers	10	--	10
Nurses	31	--	31
ASHAs	30	30	--
Private:			
GPs with degree (MBBS)	32	15	17
GPs without degree (no MBBS)	31	16	15
Pharmacy staff, total	121	60	61
Owners/managers	61	30	31
Assistants	60	30	30
**KENYA**	**Total**	Rural	Urban
Health care workers, total (public and private)	117	72	45
Public:			
Clinical officers	45	27	18
Nurses	45	27	18
Private: doctors	27	18	9
Pharmacy staff, total:	144	80	64
Owners/managers	74	42	32
Pharmacy assistants	70	38	32

Notes: interviews were spread across 4 states in India (Uttar Pradesh, Tamil Nadu, Maharashtra, West Bengal) and 4 provinces in Kenya (Nairobi, Nyanza, Rift Valley, Coast). India abbreviations: ASHA (Accredited Social Health Activist); GP (General Practitioner); MBBS (Bachelor of Medicine, Bachelor of Surgery).

Table s2: acceptable speed of action	INDIA	KENYA
Acceptable speed of action:	(N = 1013)	(N = 1001)
Less than one full day	30 %	30 %
One full day	19 %	23 %
One-and-a-half to two days	28 %	28 %
Three days or longer	23 %	19 %
**Mean desired time (days) to recovery**	**1.7**	**1.6**
Standard deviation	1.2	1.07
Confidence interval (95%)	1.6-1.8*	1.5-1.7*
Note: carers data from R1 and R2 surveys, equally weighted. *At 95% CI.		

Table s3. Reasons given for ranking antibiotics or ORS first (for ranking results see Table 3)

Ranking for:	Antibiotics: why ranked 1st (top mentions)		ORS: why ranked 1st (top mentions)	
	**INDIA**	**KENYA**	**INDIA**	**KENYA**
**Most effective** at treating the cause of diarrhea	(N = 211)<br>Reduce frequency of bowel movements (52 %);<br>Gives quick relief (22 %);<br>Stops diarrhea immediately (12%)	(N = 181)<br>Stops diarrhea … (51 %)<br>…quickly (33 %);<br>Kills bacteria/germs (27 %)	(N = 101)<br>Hardens the stool (29 %);<br>Reduces frequency of bowel movements (25 %);<br>Improves energy (23 %)	(N = 140)<br>Stops diarrhea… (58 %)<br>…quickly (33 %);<br>Rehydrates (25 %);<br>Improves energy (11 %)
**Strongest medicine** for diarrhea	(N = 252)<br>Stops diarrhea immediately (35 %);<br>Stops diarrhea quickly (20 %);<br>Any speed of action (72 %)	(N = 222)<br>Stops diarrhea (36 %)<br>Kills bacteria/germs (14 %);<br>Trusts antibiotics (12 %)	(N = 93)<br>Speed of action (40 %);<br>Replenishes lost fluids (28 %);<br>Improves energy (24 %)	(N = 117)<br>Stops diarrhea (40 %);<br>Replenishes fluid (15 %);<br>Improves energy (15 %)
**Easiest** to get children to take	(N = 87)<br>Easy to give (67 %);<br>Easy to prepare (9 %)	(N = 140)<br>Sweet taste (31 %);<br>Good taste (24 %);<br>Comes in syrup form (14 %)	(N = 126)<br>Easy to give to children (47 %);<br>Good taste (44 %)	(N = 157)<br>Good taste (33 %);<br>Sweet (25 %);<br>Is given in liquid form (15 %)
**Best use of money** to treat diarrhea	(N = 294)<br>Stops diarrhea immediately (36%)<br>Affordable (13%)	(N = 199)<br>Affordable (42 %);<br>Effective (21 %);<br>Speed of action (17 %)	(N = 110)<br>Affordable (38 %);<br>Easy for children to take (21 %);<br>Stops diarrhea immediately (15 %)	(N = 203)<br>Affordable (53 %);<br>Free (20 %);<br>Effective (14 %)

Notes: R2 survey. *Used at last episode of diarrhea.

Table s4: views from pharmacy staff and health care workers								
	INDIA				KENYA			
	Pharmacy staff*		Health care workers		Pharmacy staff*		Health care workers	
	N = 61 (below are %)		N = 134 (below are %)		N = 74 (below are %)		N = 117 (below are %)	
Statements:	Agree	Disagree	Agree	Disagree	Agree	Disagree	Agree	Disagree
Mothers like to be given real medicines.	98 %	2 %	97 %	3 %	91 %	9 %	95 %	4 %
Mothers like to be given the most powerful treatment for diarrhea.	82 %	18 %	84 %	16 %	86 %	14 %	90 %	9 %
Antibiotics are the most effective treatment for diarrhea.	74 %	25 %	74 %	19 %	57 %	42 %	25 %	74 %
Mothers do not really understand that ORS rehydrates the child.	36 %	61 %	39 %	54 %	55 %	41 %	54 %	45 %

Note: 'agree' and 'disagree' do not sum to 100% due to a few 'don't know' responses (not shown). *Only owners/managers were asked for this exercise.

Table s5: treatments given at last episode of diarrhea ~ India detail by socioeconomic grade (see also Table 5, treatments given at last episode of diarrhea)

	ever-users of ORS				never users, but aware of ORS			
Socioeconomic grades: A-E urban, R1-4 rural	A,B,C1	C2,D,E	R1,R2	R3,R4	A,B,C1	C2,D,E	R1,R2	R3,R4
Main treatments given:	(N = 228)	(N = 37)	(N = 66)	(N = 193)	(N = 185)	(N = 51)	(N = 47)	(N = 108)
Antibiotics	45 %	57 %	63 %	73 %	49 %	49 %	55 %	60 %
ORS	89 %	95 %	96 %	91 %	--	--	--	--
SSS	67 %	68 %	44 %	50 %	75 %	66 %	54 %	50 %
Other home remedy	22 %	19 %	24 %	9 %	18 %	10 %	9 %	9 %

Combined results from R1 and R2 surveys, weighted equally. Kenya detail by socioeconomic grade not included due to lack of notable differences between grades. Socioeconomic grades: ABC1 middle to high grades; C2DE lower to lower-middle grades; R1R2 rural middle to high grades; R3R4 rural lower to lower-middle grades.

Table s6. Duration of last diarrhea episode, caregiver reaction time, and timing of treatment administration

	INDIA		KENYA	
Duration of last episode of diarrhea:	Ever-users of ORS (N = 529)	Never-users but aware of ORS (N = 484)	Ever-users of ORS (N = 534)	Never-users but aware of ORS (N = 467)
1 day	2 %	3 %	7 %	9 %
2 – 4 days*	62 %	64 %	64 %	66 %
5 – 7 days*	30 %	32 %	25 %	21 %
8 or more days	5 %	3%	4 %	3 %
Mean duration, in days (standard deviation)	4.3 (2.3)	4.0 (2.0)	4.2 (3.5)	3.7 (2.5)
Median duration, in days (IQR)	4 (2)	3(3)	3 (3.2)	3 (2)
Reaction time at last episode of diarrhea:				
Carer took any action as soon as they noticed diarrhea	75 %	(N = 1013)	60 %	(N = 1001)
Median waiting time, among those who waited:	1 full day	(N = 253)	1 full day	(N = 400)
No treatment at last episode, before diarrhea ceased	N = 5	N = 20	N = 40	N = 104

	INDIA			KENYA		
	Antibiotics	ORS	SSS	Antibiotics	ORS	SSS
Treatment administration: day started, days continued	N = 313	N = 284	N = 302	N = 252	N = 212	N = 135
Started (median, day of episode): (IQR)	Day 2(1)	Day 2 (1)	Day 1 (0)	Day 2(2)	Day 2(1)	Day 1(1)
Duration of administration (median): (IQR)	3 days (2)	3 days (2)	3 days(1.5)	4 days(2)	3 days(1)	2 days(1)

Notes: R1 and R2 surveys, equally weighted, for taking action and waiting time; round 1 only for treatment administration day started, days continued.
*Grouped data (e.g., '2-4 days' combines responses for 2, 3, and 4 days). Treatment administration at last episode of diarrhea.

Table s7: primary sources of main treatments used at last episode of diarrhea, and whether recommended

	INDIA		KENYA	
	Antibiotics	ORS	Antibiotics	ORS
Where obtained:	N = 313	N = 284	N = 252	N = 212
Private	80 %	80 %	27 %	21 %
Public	19 %	18 %	48 %	67 %
Recommended*	95 %	75 %	77 %	89 %

Notes: R1 survey. *Recommended by healthcare professional or pharmacy/drugstore staff.

Table s8. Preferred presentation form, pack size	INDIA	KENYA
Preferences for presentation form: most preferred choice*	(N = 609)	(N = 600)
Conventional sachet	41 %	21 %
Ready-to-drink, box	23 %	21 %
Ready-to-drink, plastic bottle	19 %	29 %
Syrup, followed by water	12 %	16 %
Tablet, self-dissolving in water	3 %	7 %
Alternative sachet	2 %	5 %
Preferences for ORS pack size: most preferred choice*		
200 mL	78 %	59 %
1 L	22 %	41 %

*R1 survey. Illustrations were shown to respondents, who then indicated their preferred choice; the presentation choices were introduced as options for ‘a new product to treat diarrhea’, and the usage of each was described to respondents using simple statements.
